# Molecular diagnostics tailoring personalized cancer therapy—an oncologist’s view

**DOI:** 10.1007/s00428-023-03702-7

**Published:** 2023-11-20

**Authors:** Jakob M. Riedl, Florian Moik, Tamara Esterl, Sarah M. Kostmann, Armin Gerger, Philipp J. Jost

**Affiliations:** 1 Division of Oncology, Department of Internal Medicine, Medical University of Graz, Graz, Austria; 2 Medical Department III for Haematology and Oncology, School of Medicine, Technical University of Munich, Munich, Germany; 3 BioTechMed-Graz, Graz, Austria

**Keywords:** Precision oncology, Molecular oncology, Targeted therapy, Personalized treatment, Cancer

## Abstract

Medical oncology is rapidly evolving with the implementation of personalized, targeted therapies. Advances in molecular diagnostics and the biologic understanding of cancer pathophysiology led to the identification of specific genetic alterations as drivers of cancer progression. Further, improvements in drug development enable the direct interference with these pathways, which allow tailoring personalized treatments based on a distinct molecular characterization of tumors. Thereby, we are currently experiencing a paradigm-shift in the treatment of cancers towards cancer-type agnostic, molecularly targeted, personalized therapies. However, this concept has several important hurdles and limitations to overcome to ultimately increase the proportion of patients benefitting from the precision oncology approach. These include the assessment of clinical relevancy of identified alterations, capturing and interpreting levels of heterogeneity based on intra-tumoral or time-dependent molecular evolution, and challenges in the practical implementation of precision oncology in routine clinical care. In the present review, we summarize the current state of cancer-agnostic precision oncology, discuss the concept of molecular tumor boards, and consider current limitations of personalized cancer therapy. Further, we provide an outlook towards potential future developments including the implementation of functionality assessments of identified genetic alterations and the broader use of liquid biopsies in order to obtain more comprehensive and longitudinal genetic information that might guide personalized cancer therapy in the future.

## Introduction

Precision oncology is currently revolutionizing the treatment of patients with cancer. Deciphering the underlying molecular alterations as drivers of cancer development and progression has led to an improved ability to understand and potentially interfere with pro-oncogenic pathophysiologic pathways [1, 2]. This development has been enabled by several key factors. First, analyzing genetic information has technically evolved in the recent years via methods of next-generation sequencing (NGS), enabling inexpensive and fast molecular diagnostics in routine clinical practice [1, 3]. Further, decades of experimental research have led to the precise characterization of distinct oncogenic pathways including pro-oncogenic driver mutations, tumor-suppressive mechanisms, and components of the cancer-host interaction including mechanisms of impaired anti-cancer immunity [4]. Third, advances in drug development have led to the ability to directly interfere with these distinct molecular pathways (e.g., via tyrosine kinase inhibitors or monoclonal antibodies), which allows the development of personalized treatment approaches based on distinct patterns of genetic alterations [5].

In the present review, we aim to define the concept of personalized cancer therapy specifically in the context of molecular diagnostics, to discuss the application of molecular profiling in tumor-agnostic therapeutic decision-making, and to point out current challenges and potential future directions of the precision oncology approach from an oncologist´s view.

## The precision oncology paradigm

By incorporating comprehensive information on an individual patient’s level, the precision medicine approach aims to effectively guide disease prevention, diagnosis, and personalized treatment selection [6]. Since cancer has been long recognized as a disease that is driven by an accumulation of genetic aberrations, the field of oncology has taken a pioneering role in the precision medicine paradigm [7, 8]. Historically, medical cancer therapy comprised a small number of cytotoxic chemotherapies that were selected depending on the respective tumor histology and disease location. Prompted by a steadily improved understanding of carcinogenesis and genetics, which was mainly enabled by the development of novel DNA analysis techniques such as polymerase chain reactions, finally in the late 1990s the first molecular targeted drug therapies were developed. The successful clinical implementation of the monoclonal HER2-antibody trastuzumab and the BCR-ABL tyrosine kinase inhibitor imatinib marked the earliest milestones in precision oncology and heralded a new treatment era of molecular stratified cancer therapy [9, 10]. In parallel, fundamental technological advances that have resulted in the development of NGS have revolutionized molecular profiling by dramatically decreasing analytic costs and turnaround time. In contrast to conventional sequencing techniques, NGS enables simultaneous analysis of multiple genes with high accuracy [11]. Due to its high efficiency, the development of NGS thus paved the way for large-scale sequencing efforts including the cancer genome atlas projects that enabled a comprehensive genomic characterization of various tumors and thereby further transformed our understanding of oncogenesis and cancer evolution [12]. Importantly, several recurrent genetic alterations were detected across different cancer types and subsequently characterized as potential therapeutic targets. Consequently, over recent years, an extensive and rapidly growing arsenal of drug therapies targeting numerous genetic alterations including gene mutations, rearrangements, and amplifications have been developed and effectively implemented in clinical practice [13]. This was accompanied by a steadily rising application of NGS technologies in routine clinical practice for tailoring molecular stratified cancer treatment decisions in various tumor types such as non-small cell lung cancer [14], colorectal cancer [15], and biliary tract cancer [16]. Finally, a significant step towards a personalized cancer treatment strategy has been taken with the recent approval of the first tumor-agnostic therapies, which are administered based merely on the discovery of a specific molecular mutation regardless of cancer histology and tissue of origin [17].

## Integration of precision oncology in patient care

Although the concept of cancer-agnostic personalized treatment guided by molecular profiling is highly promising, its successful clinical implementation is accompanied by key challenges that require careful consideration. One major practical hurdle is the complex and multistep workflow of matching targeted therapies to detected molecular alterations [18]. This process starts by defining the questions whether the overall health status of the patient allows for implementation of molecular profiling, at what time point during the patient’s journey molecular profiling is initiated, whether a re-biopsy of the tumor lesion or, alternatively, a liquid biopsy is needed, and, last but not least, which diagnostic genetic analysis should be conducted. Further steps include NGS-analysis and bioinformatic data processing, variant calling, and the functional assessment of identified genetic alterations, parameters which are coordinated by pathologists and geneticists [19]. Each individual step in this multi-layered process poses specific challenges and pitfalls that are discussed in more detail in other articles of this series.

Yet, from an oncologist’s view, we consider the final step of the precision oncology workflow, namely the clinical annotation and clinical actionability assessment of detected molecular alterations, as the most critical and least defined step in the implementation of precision oncology.

**Fig. 1:**
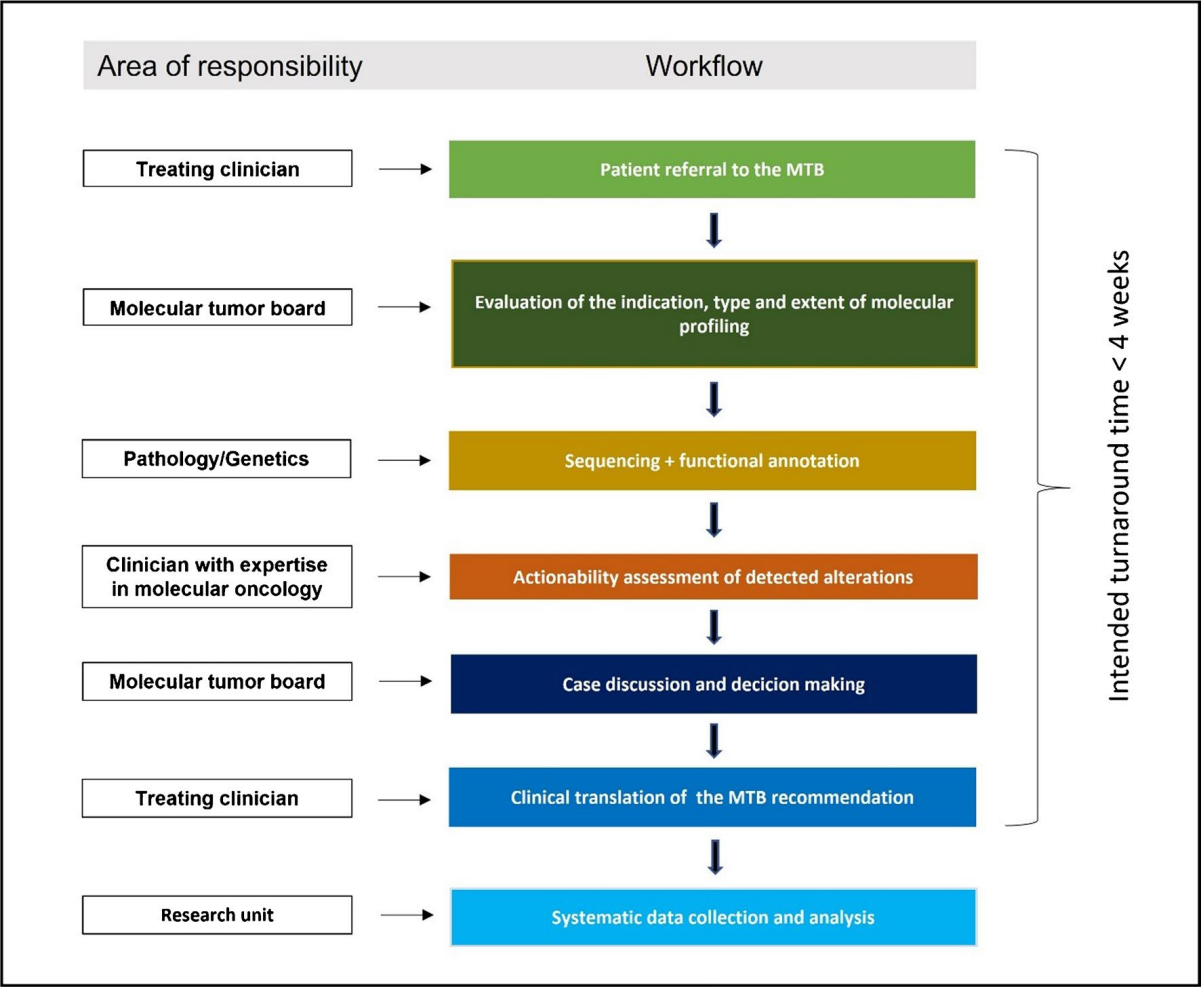
Precision oncology workflow according to a standardized Molecular Tumor Board at the university hospital of the Medical University of Graz

In this review, we will focus on the clinical remits of personalizing cancer therapies based on individual genetic compositions, pointing out key aspects including the purposeful selection of patients suitable for extended profiling, decision criteria for the choice of diagnostics, and finally the process of actionability assessment and biomarker guided therapeutic decision-making. Figure 1 illustrates the workflow of a highly standardized and outcome-centered molecular tumor board (MTB) at a major Austrian academic center, which might serve as a potential template for integrating genomic cancer sequencing in clinical care among others.

## Who shall we test, when shall we test, how shall we test?

Currently, the overall efficacy of identifying actionable targets by molecular profiling in unselected patients with cancer remains low [20]. Therefore, the European Society for Medical Oncology (ESMO) restricts its recommendations on the routine clinical use of multigene NGS testing to advanced non-small-cell lung cancer, prostate cancer, ovarian cancer, and cholangiocarcinoma. In advanced colorectal cancer, multigene testing can be considered an alternative to single gene polymerase chain reaction testing if additional costs are acceptable [21]. Apart from that, multigene sequencing to tailor genome-guided individualized therapies is not routinely recommended and should only be performed within the framework of an academic program and restricted to patients in whom the testing results might have a direct impact on the clinical management [22]. In contrast, major academic centeres in the USA and elsewhere opt for early and comprehensive tumor and germline genetic testing in largely all cancer patients [23], a view that remains not uncontested [24].

The first critical step of genomic cancer sequencing remains the careful clinical evaluation whether molecular profiling is even indicated. In general, at the current level of understanding of tumor biology and available targeted therapies, it is unreasonable and ineffective to perform comprehensive genomic profiling in every early-stage cancer, as highly efficacious established treatments might be available in this setting [22]. On the other hand, patients with advanced cancers considered for molecular profiling must be candidates to receive further antineoplastic treatment based on performance status, comorbidities, organ functions, and patient preference. Genomic profiling in patients with a reduced performance status or significant comorbidity burden are less likely to benefit from targeted therapies. Therefore, pursuing precision oncology approaches in such patients might even be detrimental as they might raise false hopes and even delay adequate palliative care interventions [25].

In any case molecular profiling is considered, the patient’s autonomy must be carefully preserved. Therefore, prior molecular profiling is initiated; it is inevitable to inform the patient accurately and comprehensively about the realistic chance of finding a potential target, as well as potential implications of somatic mutational tumor testing such as the detection of molecular alterations highly suspicious for inherited cancer syndromes.

The most robust clinical data for the implementation of extended molecular profiling to tailor targeted therapy exists for patients who have exhausted all established and clinically efficacious treatment options and retain an adequate performance status. In this setting, several precision oncology trials evaluating different concepts of NGS testing to guide targeted cancer treatment have reported promising results [26–29]. Furthermore, patients with rare cancers with limited evidence-based treatment options and patients with exceptional treatment response patterns are potentially favorable candidates for extended genetic profiling [30, 31]. However, it remains an open question whether early initiation of precision oncology might provide more benefit to cancer patients [32]. This is based on the hypothesis that targeted drug exert better effects in cancer cells prior to their exposure to several lines of chemotherapy, radiotherapy, or alternative treatment modalities.

In current clinical practice, targeted cancer gene hotspot panels covering 20–500 genes are mostly used for genomic profiling. In this regard, various NGS platforms are available each offering a slightly different spectrum of DNA and RNA coverage [22]. The choice of panel sequencing must be made individually depending on several factors including the type and stage of disease, the treatment history, the availability of previous sequencing results, the accessibility of targeted treatments, and of course the financial resources [4]. At present, the use of comprehensive genomic profiling including whole genome, whole exome, and transcriptome sequencing is mainly restricted to scientific purposes. The benefit for patient outcomes when comparing large comprehensive genomic sequencing efforts to targeted cancer gene panels is likely, but so far, the data suggesting a robustly improved identification of clinically relevant somatic alterations remains inconclusive. Several trials evaluating the clinical utility of comprehensive genomic profiling are currently ongoing [33].

Another remaining controversial aspect of molecular profiling in clinical practice is whether archival tissue should be used for profiling. Considering that most patients who are candidates for extended genomic profiling have advanced disease in which clonal evolution is known to take place under therapeutic selective pressure, most precision oncology trials involve obligatory fresh biopsies to screen for molecular targets [29, 34]. However, in clinical routine, obtaining invasive procedures of tissue re-biopsies poses significant challenges based on patient preference, procedural risk, and issues of time delay. Since genomic profiling of matched tissue and circulating tumor DNA (ctDNA) samples have shown high concordance rates of detected molecular aberrations, minimally invasive liquid biopsy of circulating tumor DNA offers an increasingly attractive alternative in this setting [35–38]. Beyond that, as ctDNA is thought to be released into the bloodstream from different tumor lesions at the same time, liquid biopsy might even provide a more comprehensive capture of the molecular composition of a patient’s tumor than a single tissue biopsy [39, 40]. However, the reliability and accuracy of ctDNA sequencing has to be further improved and clinically validated before a widespread implementation for tailoring genome guided treatment decisions can be performed in routine clinical practice [41].

## Therapeutic actionability assessment of molecular alterations

The work process of actionability assessment heavily relies on the accuracy and validity of the molecular report provided by the pathologist. Hence, a state of the art functional annotation and appropriate reporting of detected alterations represents a key prerequisite for all further steps of actionability assessment [19].. This underlines the crucial role of pathologists and the necessity of close interdisciplinary interaction between clinicians and pathologists for the successful implementation of precision oncology in clinical care. Since the functional role of variants of unknown significance is unclear, only variants classified as pathogenic or likely pathogenic are usually considered for the actionability assessment. The identification of predictive biomarkers for antineoplastic therapy represents the cornerstone of the clinical annotation process. So far, a broad and steadily increasing spectrum of molecular predictive biomarkers could be identified and clinically validated in specific cancer types. These include gene amplifications and protein overexpression (e.g., HER2) [10], gene mutations (e.g., BRAFV600E) [42]., gene fusions (e.g., EML4-ALK rearrangement)[43], and compound biomarkers such as the microsatellite status [44] and tumor mutational burden [45].. Many of these genetic drivers can be found across multiple cancer types with varying frequencies, which prompted the idea of genome guided treatment selection irrespective of the cancer histology and origin. Although this concept is highly promising and has been recently reinforced by the paramount example of highly efficacious tumor agnostic NTRK fusion targeting [46], we have been taught that the efficacy of targeted therapies in one cancer type cannot be automatically extrapolated to others. This is perfectly illustrated by the BRAF V600E mutation that can be effectively targeted by single agent or combined BRAF plus MEK inhibition in metastatic melanoma [42]. and NSCLC [47], but not in colorectal cancer due to a feedback upregulation of the epidermal growth factor receptor (EGFR) that necessitates further EGFR blockade [48]. Hence, the key challenge of the actionability assessment is to interpret the detected molecular alteration in the context of the present cancer histology and the co-mutational tumor profile.

For this purpose, a comprehensive literature research of preclinical and clinical evidence is critical. Fortunately, various partly publicly available precision oncology knowledge databases such as OncoKB, the Jackson Laboratory Clinical Knowledge Base, and My Cancer Genome that provide regularly updated curated data on cancer associated molecular alterations including reference to their clinical actionability have been established [49]. Furthermore, commercially available decision support platforms that utilize different algorithms of molecular profiling guided therapy matching are offered. Importantly, the concordance of the actionability assessment by these platforms has been shown to be rather low [50], which underlines that algorithm generated treatment suggestions should only be used for decision support and must always be critically scrutinized by a clinician in the context of the individual patient case. Accordingly, it will be necessary that specialized oncological centers with sufficient infrastructural support provide the clinical annotation of molecular profiles for individual patients because treatment decisions based solely on decision support software fail to yield a comprehensive overview of therapeutic options [50].

To harmonize the clinical interpretation and actionability assessment of molecular alterations for personalized cancer treatment, the European Society for Medical Oncology Translational Research and Precision Medicine Working Group has proposed a framework that shall enable a more precise classification and prioritization of molecular targets. With due regard to the available clinical evidence supporting a biomarker drug interaction and its consequent clinical implications, the ESMO Scale for Clinical Actionability of molecular Targets (ESCAT) defines six levels of evidence for molecular targets [51]. Tier I comprises alteration drug matches that have been shown to result in improved clinical outcome in prospective clinical trials. According to the underlying trial design, further subclassifications of the evidence level in Tier Ia (randomized), Tier Ib (non-randomized), and Tier Ic (basket trial) are feasible. Tier I targets should be considered standard of care. Tier II defines alteration drug matches that are associated with clinical activity; however, the magnitude of benefit is not clear yet. These are considered investigational targets, which should be primarily matched within the framework of a clinical trial or registry study. Tier III describes hypothetical alteration drug matches, which are suspected to result in a potential clinical benefit based on prospective trial data on the same target in another cancer type (Tier IIIa) or on the detection of an alteration functionally closely related to a known Tier I alteration (Tier IIIb). Tier III targets should be ideally investigated as part of innovative precision oncology trial concepts such as N-of-1 trials. Tier IV targets are supported exclusively by preclinical evidence and should thus not be considered targetable in clinical practice. Tier V alteration drug matches have been shown to be associated with antitumor activity that however did not translate into improved survival. In this regard, combinational therapy approaches can be considered within the framework of a clinical trial if functionally plausible. Tier X alterations have no preclinical or clinical evidence of actionability.

## Role of the molecular tumor board in personalized cancer therapy

Owing to the rapidly increasing number of already established and newly identified molecular biomarkers and corresponding approved targeted therapies, a pertinent clinical interpretation of genomic sequencing results has lately become increasingly complex and time consuming. Genomic knowledge databases and decision support platforms outlined previously can assist in the clinical actionability assessment of detected alterations; most clinicians are neither aware of these tools nor do they have the genetic knowledge and timely resources for an accurate interpretation of the literature. Therefore, an expert evaluation of sequencing results is crucial to optimize the efficient clinical application of NGS testing for therapeutic target identification. For this purpose, molecular tumor boards are increasingly established in cancer centers, which provide a multidisciplinary platform to enable the successful integration of the precision oncology approach in patient care. So far, universal recommendations on the composition and workflows of molecular tumor boards (MTBs) are missing. However, most MTBs are constituted by experts from different medical fields including clinicians, pathologists, geneticists, bioinformaticians, and molecular biologists. The main tasks of the MTB encompass the initiation of appropriate genetic testing, assessment of molecular profiling results for target identification and personalized treatment recommendation, assistance of diagnosis in cases with indeterminate histology aberrations, and the detection of inherited cancer susceptibilities [8, 52].

To ensure optimal decision-making, a comprehensive review and evaluation of the medical history, the duration and response of previous antineoplastic therapies, and the availability of archival tumor samples, as well as previous molecular testing results of each individual patient is inevitable.

In addition to the abovementioned areas of responsibility, the MTB has a key educational role to deepen the understanding of molecular oncology and spread the knowledge how to adequately utilize cancer genome diagnostics for tailoring patient care. Further, MTBs shall serve as a venue to foster innovative translational research projects with the ultimate goal of identifying novel predictive biomarkers and resistance mechanisms and thereby fully incorporating the research concept of bedside-to-bench and back [53].

Since the appropriate implementation of MTBs requires a high level of expertise from different medical specialties that are usually only provided by selected academic institutions, so far only a small minority of cancer patients can benefit from MTB facilities [54]. This poses one key challenge, which might be overcome by the implementation of centrally coordinated precision oncology initiatives that provides a virtually accessible platform for patient case discussion, knowledge exchange, and translation research design across multiple cancer institutions [55].

## Tumor-agnostic genomic targets as blueprints for the precision oncology paradigm

The identification of tumor-agnostic unifying molecular compositions that enable personalized therapies is the main goal in precision oncology. In recent years, multiple genetic alterations have been identified that serve as therapeutic targets irrespective of underlying cancer types (Table 1). In the following section, we provide a brief overview of two established examples of tumor-agnostic genomic targets as prime examples for the high therapeutic potential of personalized oncology approaches.

**Table 1:** Overview of molecular targets with approved biomarker guided therapies in solid cancers. *FISH* fluorescence in situ hybridization, *IHC* immunohistochemistry, *NSCLC* non-small-cell lung cancer

Target	Type of alteration	Method of testing	Approved drugs	Clinical indication*
ALK	Gene fusion	RNA sequencing IHC screening	Alectinib Brigatinib Ceritinib Crizotinib Lorlatinib	NSCLC
BRAF	Mutation	DNA sequencing	Dabrafenib Encorafenib Vemurafenib	Anaplastic thyroid carcinoma Colorectal cancer Malignant melanoma NSCLC
BRCA	Mutation	DNA sequencing	Niraparib Olaparib Platinum chemotherapy Rucaparib Talazoparib Veliparib	Breast cancer Ovarian cancer Prostate cancer
EGFR	Mutation	DNA sequencing	Amivantamab Erlotinib Gefitinib Osimertinib	NSCLC
ERBB2	Overexpression Amplification Mutation	IHC FISH DNA sequencing	Lapatinib Neratinib Pertuzumab Trastuzumab Trastuzumab-emtansine Trastuzumab-deruxtecan	Breast cancer Colorectal cancer Esophageal cancer Gastric cancer NSCLC
FGF(R)	Mutation Gene fusion	DNA sequencing RNA sequencing	Erdafitinib Futibatinib Pemigatinib	Biliary tract cancer Urothelial cancer
Homologous recombination deficiency	Genomic instability	DNA sequencing	Niraparib Olaparib Platinum chemotherapy Rucaparib Talazoparib Veliparib	Ovarian cancer Prostate cancer
KIT	Mutation	DNA sequencing	Imatinib	GIST
MET	Amplification Mutation	FISH DNA sequencing	Cabmatinib Tepotinib	NSCLC
Microsatelitte instability / Mismatch repair deficiency	Genomic instability	DNA sequencing IHC	Pembrolizumab	Tumor agnostic
NTRK	Gene fusion	RNA sequencing IHC screening	Entrectinib Larotrectinib	Tumor agnostic
PDGF(R) A	Mutation	DNA sequencing	Avapritinib	GIST
PD-L1	Overexpression	IHC	Atezolizumab Cemiplimab Durvalumab Nivolumab Pembrolizumab	Breast cancer (triple negative) Cervical cancer Esophageal cancer Gastric cancer Head and neck cancer NSCLC Urothelial cancer
PIK3CA	Mutation	DNA sequencing	Alpelisib	Breast cancer
RET	Fusion	RNA sequencing	Pralsetinib Selpercatinib	NSCLC Thyroid cancer
ROS	Fusion	RNA sequencing	Crizotinib Entrectinib Lorlatinib	NSCLC
Tumor mutational burden	Genomic instability	DNA sequencing	Pembrolizumab	Tumor agnostic

*Biomarker guided

## Genetic hypermutability and microsatellite instability

Microsatellite instability and genetic hypermutability have lately received increasing clinical attention as tumor-agnostic predictive biomarkers for immune checkpoint inhibitor response. Microsatellite instability is caused by DNA mismatch repair deficiency that evokes an accumulation of genetic alterations in short non-coding repetitive DNA segments distributed throughout the genome and referred to as microsatellites. Since the DNA mismatch repair system plays a key role in maintaining genomic stability, its deficiency is further associated with an increased number of somatic tumor mutations [56]. The phenomenon of genetic hypermutability specified by the tumor mutational burden (TMB) strongly correlates with the abundance of tumor neoantigen formation which has been proposed to be critical for immune checkpoint inhibitor (ICI)–mediated T cell response [57]. These findings prompted the clinical investigation of ICI therapy in patients with DNA mismatch repair deficiency and/or high TMB. DNA mismatch repair deficiency that can be either assessed on the protein expression level by immunohistochemistry or indirectly by the genomic detection of microsatellite instability (MSI) can be found across various cancer types with an overall prevalence of approximately 4%. The highest disease-specific prevalence of MSI is observed in Lynch syndrome–associated cancers including endometrial, colorectal, and gastric adenocarcinoma [58]. Importantly, in a seminal study by Le et al., PD-1 blockade with the ICI inhibitor pembrolizumab resulted in a remarkable response rate of 52% and a high proportion of durable remissions in heavily pre-treated patients with different types of MSI high advanced carcinomas [59]. These findings prompted the first tumor-agnostic therapy approval by the Food and Drug Administration (FDA), which was recently further justified by several cancer type specific trials confirming the exceptional efficacy of ICI therapy in patients with MSI high tumors [60]. The tumor-agnostic predictive validity of the TMB is less clear. One basket phase II trial enrolling patients with selected advanced solid tumors, demonstrated a significantly higher response rate of the PD-1 antibody pembrolizumab in patients with TMB high tumors, defined as ≥ 10 tumor specific mutations/megabase detected by the targeted FoundationOne CDx assay [61]. While data on major tumor types such as breast, colorectal, and prostate cancer not included in this trial were missing, the FDA approved pembrolizumab for treatment of TMB high tumors irrespective of cancer histology. This approval was challenged by a comprehensive retrospective cohort study of more than 1500 patients treated with ICI therapy by McGrail et al. indicating that the TMB only discriminates ICI efficacy in the subset of tumor types in which CD8 cells correlate with the neoantigen load, whereas in other cancer types such as breast and prostate cancer the TMB was not associated with ICI response. Importantly, tumor specific subgroups in this study were small, which alleviates its general validity [62]. Thereby, further research is warranted to clarify the tumor-agnostic predictive role of the TMB for ICI efficacy.

## NTRK fusion

Neurotrophic tropomyosin-receptor kinase (NTRK) genes are physiologically involved in neural development and encode a family of receptor tyrosine kinases [63]. Fusions of NTRK genes have been identified as oncogenic drivers in different solid and hematologic malignancies in adults and children, with heterogeneous gene fusion partners that constitutively activate tyrosine kinase signalling [46]. In general, NTRK fusions represent a very rare type of genetic alterations in general oncologic populations. For example, two large genetic screening studies suggest an overall prevalence of NTRK-fusions of 0.3% among cancer patients [64, 65]. However, in several rare cancers including secretory breast carcinoma, infantile fibrosarcoma, secretory salivary gland cancer, or pediatric thyroid carcinomas NTRK fusions represent a common genetic alteration [65]. Further, albeit rare, NTRK fusions are detected across various more frequent cancers, broadening their clinical relevancy [46, 63]. Importantly, the development of tyrosine kinase inhibitors (TKIs) targeting NTRK represents a promising therapeutic approach for patients. In detail, larotrectinib and entrectinib have demonstrated profound clinical activity in the treatment of patients with cancers and underlying NTRK fusions. In a pooled analysis of basket trials enrolling patients with NTRK-fusion positive advanced cancers, larotrectinib treatment led to an ORR of 78% and median PFS was 37 months [66]. Further, ORR with entrectinib was 61%, with a median PFS of 14 months [67]. Synoptically, the availability of highly active NTRK inhibitors highlights the potential of broadly assessing NTRK fusion positivity in patients with advanced cancers beyond established therapeutic options, especially in those with specific rare cancer types [68].

## Challenges and prospect of precision oncology

Despite remarkable advances in precision oncology, important limitations and challenges of genome guided therapy remain to be solved to enable a broader and more efficient clinical implementation and thereby maximize patient benefit [2]. First, over the process of clonal evolution in tumorigenesis and cancer progression, cancers acquire a variety of pro-oncogenic molecular aberrations. Thereby, over the course of disease, cancers evolve towards a higher level of heterogeneity and subclonality [69]. Consequently, therapeutic efficacy in very advanced cancer settings is limited a priori due to the high probability of underlying genetic properties of cancers to circumvent single-target personalized therapies. Conceptually, targeting of specific cancer driver genes in earlier treatment settings might therefore enable a more pronounced anti-cancer effect. In the future, earlier integration of personalized treatment approaches in clinical care might therefore yield more promising therapeutic efficacy.

Secondly, precision oncology is currently limited in our current understanding of attributing the degree of pathogenicity of identified genetic alterations. Specifically, cancers frequently attain a number of passenger co-mutations which are not vital for cancer progression.

Furthermore, healthy tissues have been demonstrated to acquire somatic mutations with varying degrees of pathologic significance. For example, somatic mutations in hematopoiesis obtained with increasing frequency with higher age are frequently detected in diagnostic evaluation of circulating tumor DNA and thereby decrease the specificity of observed mutational patterns.

Moreover, mutations in various classic prooncogenic driver genes have been detected in different non-malignant diseases [70]. These limitations might be overcome in the future with the implementation of personalized modeling of the functional impact of identified genetic alterations and respective therapeutic targeting on RNA, protein, or cellular levels [71]. In addition, with the advent of artificial intelligence–based technologies, pathologic and clinical annotation of molecular diagnostics might be further facilitated.

Thirdly, from a practical point of view, precision oncology is currently limited in several aspects of structural and technical restraints. The current timeframe from initiation of molecular diagnostics until the actual implementation of personalized therapies might take up to several weeks. Thereby, in a mostly advanced oncologic therapeutic setting, a considerable proportion of patients are lost during the process. In addition, availability of recent tissue samples is frequently necessary to enable reliable genetic information on the current molecular makeup of a cancer, due to the process of clonal evolution and genetic mechanisms of treatment resistance that might accumulate during previous anti-cancer therapies. Therefore, personalized oncology frequently depends on the performance of novel tissue sampling and biopsy, which might affect the risk–benefit ratio of this treatment approach. However, in the future, advances in the field of liquid biopsies via analyzing circulating tumor DNA hold the potential to potentially replace the need for additional tissue-based testing [72].

Finally, one major hurdle for a widespread global adoption of the precision oncology approach that must be considered is the financial burden coming along with comprehensive genomic sequencing and even more the cost of targeted therapy itself. Unfortunately, access to molecular profiling and personalized cancer therapy is currently restricted to a small minority of patients with cancer in high-income countries. However, on the long term, more precise and efficacious treatment selection of targeted cancer therapies by improved prediction of treatment benefit might even reduce costs compared to unguided conventional treatment by enabling an ambulatory management and avoid hospitalizations associated with disease complications [73]. Studies that specifically include cost-effectiveness evaluations of the precision oncology approach are therefore urgently needed.

## Conclusion

Treatment of patients with cancer is currently undergoing a dramatic shift towards personalized therapy using molecular diagnostics. However, important limitations remain to be solved in order to maximize patient benefit, including levels of cancer-specific genetic heterogeneity, interpretation and clinical annotation of identified genetic alterations, and current technical constraints in molecular diagnostics. In the future, with an improved understanding of the complex underlying molecular mechanisms via integrating various layers of genetic and functional analyses in a refined process of personalized clinical decision-making, alongside with an enhanced ability to dynamically detect and monitor individual cancer-driving molecular aberrations via liquid biopsies, personalized oncology will dramatically change our current concept of cancer therapy.

## References

[1] Malone ER, Oliva M, Sabatini PJB, Stockley TL, Siu LL (2020). Molecular profiling for precision cancer therapies. Genome Med.

[2] Wahida A, Buschhorn L, Fröhling S (2022). The coming decade in precision oncology: six riddles. Nat Rev Cancer.

[3] Brown NA, Elenitoba-Johnson KSJ (2020). Enabling precision oncology through precision diagnostics. Annu Rev Pathol.

[4] Mateo J, Steuten L, Aftimos P (2022). Delivering precision oncology to patients with cancer. Nat Med.

[5] Dugger SA, Platt A, Goldstein DB (2018). Drug development in the era of precision medicine. Nat Rev Drug Discov.

[6] Collins FS, Varmus H (2015). A new initiative on precision medicine. N Engl J Med.

[7] Tsimberidou AM, Fountzilas E, Nikanjam M, Kurzrock R (2020). Review of precision cancer medicine: evolution of the treatment paradigm. Cancer Treat Rev.

[8] van der Velden DL, van Herpen CML, van Laarhoven HWM (2017). Molecular tumor boards: current practice and future needs. Ann Oncol.

[9] Druker BJ, Talpaz M, Resta DJ (2001). Efficacy and safety of a specific inhibitor of the BCR-ABL tyrosine kinase in chronic myeloid leukemia. N Engl J Med.

[10] Slamon DJ, Leyland-Jones B, Shak S (2001). Use of chemotherapy plus a monoclonal antibody against HER2 for metastatic breast cancer that overexpresses HER2. N Engl J Med.

[11] Berger MF, Mardis ER (2018). The emerging clinical relevance of genomics in cancer medicine. Nat Rev Clin Oncol.

[12] Weinstein JN, Collisson EA, Mills GB (2013). The Cancer Genome Atlas Pan-Cancer analysis project. Nat Genet.

[13] Waarts MR, Stonestrom AJ, Park YC, Levine RL (2022). Targeting mutations in cancer. J Clin Invest.

[14] Planchard D, Popat S, Kerr K, Novello S, Smit EF, Faivre-Finn C, Mok TS, Reck M, Van Schil PE, Hellmann MD, Peters S; ESMO Guidelines Committee (2018) Metastatic non-small cell lung cancer: ESMO clinical practice guidelines for diagnosis, treatment and follow-up. Ann Oncol 29(Suppl 4):iv192–iv237. 10.1093/annonc/mdy275. Erratum in: Ann Oncol. 2019 May; 30(5):863–870 10.1093/annonc/mdy275 30285222

[15] Gutierrez ME, Price KS, Lanman RB, Nagy RJ, Shah I, Mathura S, Mulcahy M, Norden AD, Goldberg SL (2019) Genomic profiling for KRAS, NRAS, BRAF, microsatellite instability, and mismatch repair deficiency among patients with metastatic colon cancer. JCO Precis Oncol 3:PO.19.00274. 10.1200/po.19.00274 10.1200/PO.19.00274 PMC7448804 32923867

[16] Lamarca A, Edeline J, Goyal L (2022). How I treat biliary tract cancer. ESMO Open.

[17] Looney A-M, Nawaz K, Webster RM (2020). Tumour-agnostic therapies. Nat Rev Drug Discov.

[18] Horak P, Leichsenring J, Goldschmid H (2022). Assigning evidence to actionability: an introduction to variant interpretation in precision cancer medicine. Genes Chromosom Cancer.

[19] Li MM, Datto M, Duncavage EJ (2017). Standards and Guidelines for the Interpretation and Reporting of Sequence Variants in Cancer: A Joint Consensus Recommendation of the Association for Molecular Pathology, American Society of Clinical Oncology, and College of American Pathologists. J Mol Diagn.

[20] Haslam A, Kim MS, Prasad V (2021). Updated estimates of eligibility for and response to genome-targeted oncology drugs among US cancer patients, 2006–2020. Ann Oncol.

[21] Mosele F, Remon J, Mateo J (2020). Recommendations for the use of next-generation sequencing (NGS) for patients with metastatic cancers: a report from the ESMO Precision Medicine Working Group. Ann Oncol.

[22] Colomer R, Mondejar R, Romero-Laorden N, Alfranca A, Sanchez-Madrid F, Quintela-Fandino M (2020) When should we order a next generation sequencing test in a patient with cancer? EClinicalMedicine 25:100487. 10.1016/j.eclinm.2020.100487 10.1016/j.eclinm.2020.100487 PMC7397394 32775973

[23] Subbiah V, Kurzrock R (2023). Universal germline and tumor genomic testing needed to win the war against cancer: genomics is the diagnosis. J Clin Oncol.

[24] Sorscher S. Do all patients diagnosed with cancer deserve germline testing? J Clin Oncol 41(24):4057–4058. 10.1200/jco.23.00710 10.1200/JCO.23.00710 37311151

[25] Colomer R, Miranda J, Romero-Laorden N, Hornedo J, González-Cortijo L, Mouron S, Bueno MJ, Mondéjar R, Quintela-Fandino M (2023) Usefulness and real-world outcomes of next generation sequencing testing in patients with cancer: an observational study on the impact of selection based on clinical judgement. eClinicalMedicine 60:102029. 10.1016/j.eclinm.2023.102029 10.1016/j.eclinm.2023.102029 PMC10248077 37304496

[26] Massard C, Michiels S, Ferté C (2017). High-throughput genomics and clinical outcome in hard-to-treat advanced cancers: results of the MOSCATO 01 trial. Cancer Discov.

[27] Sicklick JK, Kato S, Okamura R (2019). Molecular profiling of cancer patients enables personalized combination therapy: the I-PREDICT study. Nat Med.

[28] Rothwell DG, Ayub M, Cook N (2019). Utility of ctDNA to support patient selection for early phase clinical trials: the TARGET study. Nat Med.

[29] Von Hoff DD, Stephenson JJ, Rosen P (2010). Pilot study using molecular profiling of patients' tumors to find potential targets and select treatments for their refractory cancers. J Clin Oncol.

[30] Horak P, Heining C, Kreutzfeldt S (2021). Comprehensive genomic and transcriptomic analysis for guiding therapeutic decisions in patients with rare cancers. Cancer Discov.

[31] Tsimberidou AM, Said R, Staudt LM, Conley BA, Takebe N (2019). Defining, identifying, and understanding “exceptional responders” in oncology using the tools of precision medicine. Cancer J.

[32] Wahida A, Buschhorn L, Fröhling S (2023). The coming decade in precision oncology: six riddles. Nat Rev Cancer.

[33] Rosenquist R, Cuppen E, Buettner R (2022). Clinical utility of whole-genome sequencing in precision oncology. Semin Cancer Biol.

[34] Massard C, Michiels S, Ferté C (2017). High-throughput genomics and clinical outcome in hard-to-treat advanced cancers: results of the MOSCATO 01 trial. Cancer Discov.

[35] Adalsteinsson VA, Ha G, Freeman SS (2017). Scalable whole-exome sequencing of cell-free DNA reveals high concordance with metastatic tumors. Nat Commun.

[36] Riedl JM, Hasenleithner SO, Pregartner G (2021). Profiling of circulating tumor DNA and tumor tissue for treatment selection in patients with advanced and refractory carcinoma: a prospective, two-stage phase II Individualized Cancer Treatment trial. Ther Adv Med Oncol.

[37] Rothwell DG, Ayub M, Cook N (2019). Utility of ctDNA to support patient selection for early phase clinical trials: the TARGET study. Nat Med.

[38] Bayle A, Belcaid L, Aldea M (2023). Clinical utility of circulating tumor DNA sequencing with a large panel: a National Center for Precision Medicine (PRISM) study. Ann Oncol.

[39] Heitzer E, Haque IS, Roberts CES, Speicher MR (2019). Current and future perspectives of liquid biopsies in genomics-driven oncology. Nat Rev Genet.

[40] Parikh AR, Leshchiner I, Elagina L (2019). Liquid versus tissue biopsy for detecting acquired resistance and tumor heterogeneity in gastrointestinal cancers. Nat Med.

[41] Kim H, Park KU (2023). Clinical circulating tumor DNA testing for precision oncology. Cancer Res Treat.

[42] Chapman PB, Hauschild A, Robert C (2011). Improved survival with vemurafenib in melanoma with BRAF V600E mutation. N Engl J Med.

[43] Kwak EL, Bang Y-J, Camidge DR (2010). Anaplastic lymphoma kinase inhibition in non–small-cell lung cancer. N Engl J Med.

[44] André T, Shiu K-K, Kim TW (2020). Pembrolizumab in microsatellite-instability–high advanced colorectal cancer. N Engl J Med.

[45] Hellmann MD, Ciuleanu T-E, Pluzanski A (2018). Nivolumab plus ipilimumab in lung cancer with a high tumor mutational burden. N Engl J Med.

[46] Cocco E, Scaltriti M, Drilon A (2018). NTRK fusion-positive cancers and TRK inhibitor therapy. Nat Rev Clin Oncol.

[47] Planchard D, Besse B, Groen HJM (2016). Dabrafenib plus trametinib in patients with previously treated <em>BRAF</em><sup>V600E</sup>-mutant metastatic non-small cell lung cancer: an open-label, multicentre phase 2 trial. Lancet Oncol.

[48] Kopetz S, Grothey A, Yaeger R (2019). Encorafenib, binimetinib, and cetuximab in BRAF V600E–mutated colorectal cancer. N Engl J Med.

[49] Li X, Warner JL (2020) A review of precision oncology knowledgebases for determining the clinical actionability of genetic variants. Mini Review. Front Cell Dev Biol 8. 10.3389/fcell.2020.00048 10.3389/fcell.2020.00048 PMC7026022 32117976

[50] Perakis SO, Weber S, Zhou Q (2020). Comparison of three commercial decision support platforms for matching of next-generation sequencing results with therapies in patients with cancer. ESMO Open.

[51] Mateo J, Chakravarty D, Dienstmann R (2018). A framework to rank genomic alterations as targets for cancer precision medicine: the ESMO Scale for Clinical Actionability of molecular Targets (ESCAT). Ann Oncol.

[52] Luchini C, Lawlor RT, Milella M, Scarpa A (2020). Molecular tumor boards in clinical practice. Trends Cancer.

[53] Subbiah V, Kurzrock R (2018). Challenging standard-of-care paradigms in the precision oncology era. Trends Cancer.

[54] Gardner B, Doose M, Sanchez JI, Freedman AN, de Moor JS (2021) Distribution of genomic testing resources by oncology practice and rurality: a nationally representative study. JCO Precis Oncol 5:PO.21.00109. 10.1200/po.21.00109 10.1200/PO.21.00109 PMC8457818 34568717

[55] Horak P, Klink B, Heining C (2017). Precision oncology based on omics data: the NCT Heidelberg experience. Int J Cancer.

[56] Li K, Luo H, Huang L, Luo H, Zhu X (2020). Microsatellite instability: a review of what the oncologist should know. Cancer Cell Int.

[57] Schumacher TN, Schreiber RD (2015). Neoantigens in cancer immunotherapy. Science.

[58] Bonneville R, Krook MA, Kautto EA (2017). Landscape of microsatellite instability across 39 cancer types. JCO Precis Oncol.

[59] Le DT, Uram JN, Wang H (2015). PD-1 blockade in tumors with mismatch-repair deficiency. New England J Med.

[60] Petrelli F, Ghidini M, Ghidini A, Tomasello G (2020). Outcomes following immune checkpoint inhibitor treatment of patients with microsatellite instability-high cancers: a systematic review and meta-analysis. JAMA Oncol.

[61] Marabelle A, Fakih M, Lopez J (2020). Association of tumour mutational burden with outcomes in patients with advanced solid tumours treated with pembrolizumab: prospective biomarker analysis of the multicohort, open-label, phase 2 KEYNOTE-158 study. Lancet Oncol.

[62] McGrail DJ, Pilié PG, Rashid NU (2021). High tumor mutation burden fails to predict immune checkpoint blockade response across all cancer types. Ann Oncol.

[63] Hechtman JF (2022). NTRK insights: best practices for pathologists. Mod Pathol.

[64] Solomon JP, Linkov I, Rosado A (2020). NTRK fusion detection across multiple assays and 33,997 cases: diagnostic implications and pitfalls. Mod Pathol.

[65] Rosen EY, Goldman DA, Hechtman JF (2020). TRK fusions are enriched in cancers with uncommon histologies and the absence of canonical driver mutations. Clin Cancer Res.

[66] McDermott R, van Tilburg CM, Farago AF (2020). 1955P Survival benefits of larotrectinib in an integrated dataset of patients with TRK fusion cancer. Ann Oncol.

[67] Demetri GD, De Braud F, Drilon A (2022). Updated integrated analysis of the efficacy and safety of entrectinib in patients with NTRK fusion-positive solid tumors. Clin Cancer Res.

[68] Marchiò C, Scaltriti M, Ladanyi M (2019). ESMO recommendations on the standard methods to detect NTRK fusions in daily practice and clinical research. Ann Oncol.

[69] Gerstung M, Jolly C, Leshchiner I (2020). The evolutionary history of 2,658 cancers. Nature.

[70] Adashek JJ, Kato S, Lippman SM, Kurzrock R (2020). The paradox of cancer genes in non-malignant conditions: implications for precision medicine. Genome Med.

[71] Letai A, Bhola P, Welm AL (2022). Functional precision oncology: testing tumors with drugs to identify vulnerabilities and novel combinations. Cancer Cell.

[72] Ignatiadis M, Sledge GW, Jeffrey SS (2021). Liquid biopsy enters the clinic — implementation issues and future challenges. Nat Rev Clin Oncol.

[73] Christofyllakis K, Bittenbring JT, Thurner L (2022). Cost-effectiveness of precision cancer medicine-current challenges in the use of next generation sequencing for comprehensive tumour genomic profiling and the role of clinical utility frameworks (Review). Mol Clin Oncol.

